# Hybrid Density Functional Study on the Photocatalytic Properties of Two-dimensional g-ZnO Based Heterostructures

**DOI:** 10.3390/nano8060374

**Published:** 2018-05-28

**Authors:** Guangzhao Wang, Dengfeng Li, Qilong Sun, Suihu Dang, Mingmin Zhong, Shuyuan Xiao, Guoshuai Liu

**Affiliations:** 1 School of Electronic Information Engineering, Yangtze Normal University, Chongqing 408100, China; wangyan6930@126.com (G.W.); dangsuihu@126.com (S.D.); 2 Department of Science, Chongqing University of Posts and Telecommunications, Chongqing 400065, China; 3 Chongqing Institute of Green and Intelligent Technology, Chinese Academy of Sciences, Chongqing 400714, China; sunqilong@cigit.ac.cn; 4 School of Physical Science and Technology, Southwest University, Chongqing 400715, China; 5 Wuhan National Laboratory for Optoelectronics, Huazhong University of Science and Technology, Wuhan 430074, China; syxiao@hust.edu.cn; 6 State Key Laboratory of Urban Water Resource and Environment, School of Environment, Harbin Institute of Technology, Harbin 150090, China; swift_ft@163.com

**Keywords:** ZnO/WS_2_, ZnO/WSe_2_, photocatalysis, hybrid density functional

## Abstract

In this work, graphene-like ZnO (g-ZnO)-based two-dimensional (2D) heterostructures (ZnO/WS${}_{2}$ and ZnO/WSe${}_{2}$) were designed as water-splitting photocatalysts based on the hybrid density functional. The dependence of photocatalytic properties on the rotation angles and biaxial strains were investigated. The bandgaps of ZnO/WS${}_{2}$ and ZnO/WSe${}_{2}$ are not obviously affected by rotation angles but by strains. The ZnO/WS${}_{2}$ heterostructures with appropriate rotation angles and strains are promising visible water-splitting photocatalysts due to their appropriate bandgap for visible absorption, proper band edge alignment, and effective separation of carriers, while the water oxygen process of the ZnO/WSe${}_{2}$ heterostructures is limited by their band edge positions. The findings pave the way to efficient g-ZnO-based 2D visible water-splitting materials.

## 1. Introduction

An increasing amount of effort has been dedicated to 2D materials for their distinctive electronic [1], optical [2,3], mechanical properties [4], and their potential applications in superconductivity [5], supercapacitors [6], lithium-ion batteries [7], solar cells [8], and photocatalysis [9]. Recently, graphene-like ZnO (g-ZnO) has been experimentally synthesized [10,11] and proven to be energetically stable by density functional theory (DFT) [12]. Though there have been many investigations [13,14,15,16] focused on the magnetism of g-ZnO, few studies exist regarding the water-splitting [17,18] of g-ZnO. As bulk ZnO-based materials are promising water-splitting photocatalysts, we may wonder about the photocatalytic activity of g-ZnO- and g-ZnO-based materials. However, the bandgap for g-ZnO is 3.25 eV [13], which results in inefficient visible light absorption and reduces the utilization of solar energy. Therefore, the electronic structure of g-ZnO should be adjusted so as to reduce the bandgap and absorb more visible light. A desired water-splitting photocatalyst should have a conduction band minimum (CBM) and a valence band maximum (VBM) above the water reduction level and the water oxidation level, respectively [19,20]. Considering the additional overpotential accompanied with overall water redox processes, the theoretical bandgap for desired water-splitting photocatalyst should be larger than 1.23 eV [19,20]. Construction of a heterojunction is a useful method to improve the photocatalytic performance of photocatalysts [21,22,23,24,25,26]. The monolayer WS${}_{2}$ (WSe${}_{2}$) has been studied as a photocatalyst; the appropriate bandgap of 1.98 (1.63) [27] eV ensures its strong ability for visible light absorption. As monolayer WS${}_{2}$ (WSe${}_{2}$) has a similar crystal structure and almost the same lattice constants compared with g-ZnO, we consider building heterostructures between g-ZnO and the WS${}_{2}$ (WSe${}_{2}$) monolayer, i.e., ZnO/WS${}_{2}$ (ZnO/WSe${}_{2}$) heterostructures.

In this article, using the hybrid density functional, the structural, electronic, and optical properties and band edge alignment of ZnO/WS${}_{2}$ and ZnO/WSe${}_{2}$ heterostructures are described and discussed to explore whether they have an efficient visible light response and photocatalytic activities. The following questions are posed: (i) Will these two heterostructures be promising water-splitting photocatalysts with an appropriate bandgap and band edge positions? (ii) Will charge separation exist between the constituent monolayers? (iii) Will these two heterostructures have an efficient absorption of visible light? (iv) Will the electronic and optical properties be changed with the application of acceptable strains?

## 2. Computational Details

The heterostructure models of ZnO/WS${}_{2}$ and ZnO/WSe${}_{2}$ are built using a 2×2 supercell of g-ZnO as a substrate to support 2×2 supercells of WS${}_{2}$ and WSe${}_{2}$ monolayers, i.e., the lattice parameters of the heterostructures are the fixed value of optimized 2 × 2 g-ZnO of a=b=6.58 Å. The calculated lattice mismatch between ZnO and WS${}_{2}$ (WSe${}_{2}$) monolayer is −3.4% (+0.3%), which is helpful for experimental preparations of ZnO/WS${}_{2}$ and ZnO/WSe${}_{2}$. In addition, a vacuum space of 18 Å is adopted to avoid the interactions between neighboring nonocomposites. The Vienna ab initio simulation package (VASP) [28] was used to perform the DFT calculations, and the Perdew-Burke-Ernzernof (PBE) [29] under generalized gradient approximation (GGA) [30] within the projected augmented wave (PAW) method [31] are utilized. The DFT-D3 [32] vdW correction by Grime is adopted to treat the weak van der Waals (vdW) interactions. An energy cutoff of 500 eV, energy convergence thresholds of 10${}^{-5}$ eV, force convergence criteria of 0.01 eV/Å, and k-points of 13 × 13 × 1 for 1 × 1 g-ZnO, WS${}_{2}$ (WSe${}_{2}$) monolayers and 7 × 7 × 1 for 2 × 2 ZnO/WS${}_{2}$ (ZnO/WSe${}_{2}$) are sufficient for calculating geometric and electronic structures. To determine electronic and optical properties more accurately, the hybrid density functional of Heyd-Scuseria-Ernzerhof [33,34] (HSE06) with a mixing coefficient of 0.25 is used. In summary, the PBE is used for structural optimizations and energy calculations, while the HSE06 is adopted for the calculation of electronic structures and optical properties. Furthermore, the valence states of O (2s${}^{2}$2p${}^{4}$), S (3s${}^{2}$3p${}^{4}$), Se (4s${}^{2}$4p${}^{4}$), Zn (3d${}^{10}$4s${}^{2}$), and W (5p${}^{6}$6s${}^{2}$5d${}^{4}$) are used to construct PAW potentials. The absorption curves are calculated from the imaginary part of the dielectric constant according to the Kramers-Kroning dispersion relation [35].

## 3. Results and Discussion

**Figure 1:**
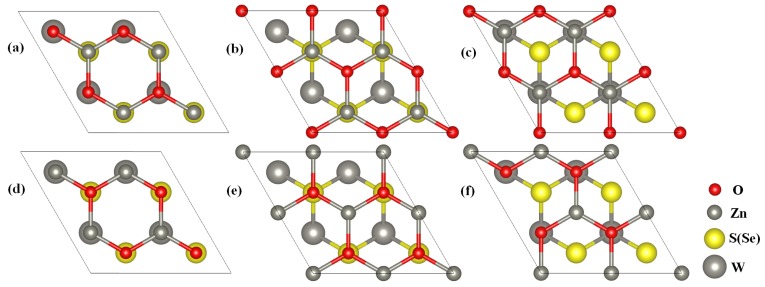
Top views of the ZnO/WS${}_{2}$ (ZnO/WSe${}_{2}$) with the g-ZnO in different rotation angles of (**a**) 0${}^{\circ }$ (the reference); (**b**) 60${}^{\circ }$; (**c**) 120${}^{\circ }$; (**d**) 180${}^{\circ }$; (**e**) 240${}^{\circ }$; and (**f**) 300${}^{\circ }$.

The obtained bandgaps for the g-ZnO and WS${}_{2}$ (WSe${}_{2}$) monolayers are, respectively, 3.30 and 2.35 (2.10) eV, consistent with previous reports [27,36]. The obtained lattice parameters for the g-ZnO and WS${}_{2}$ monolayers are 3.290 and 3.180 (3.300) Å, respectively. The lattice mismatch between the g-ZnO and WS${}_{2}$ WSe${}_{2}$ of −3.4% (0.3%) is small, which is favorable for the construction of a ZnO/WS${}_{2}$ (ZnO/WSe${}_{2}$) heterostructure. To build the ZnO/WS${}_{2}$ and ZnO/WSe${}_{2}$ heterostructure models, six different ZnO single-layers rotating on the fixed WS${}_{2}$ and WSe${}_{2}$ monolayers from 0 to 300${}^{\circ }$ with 60${}^{\circ }$ apart are considered. Top views of different stacked ZnO/WS${}_{2}$ and ZnO/WSe${}_{2}$ heterostructures are depicted in Figure 1. The Zn–O bond lengths in all these ZnO/WS${}_{2}$ and ZnO/WSe${}_{2}$ heterostructures are the same value of 1.900 Å, which is easy to understand because the lattice parameters of these heterostructures are the fixed values of the 2 × 2 g-ZnO and WS${}_{2}$ layer (WSe${}_{2}$) hardly affects the ZnO layer in the composites because of the weak vdW interactions. The lengths of the W–S bond in ZnO/WS${}_{2}$ with rotation angles in the range of 0–300${}^{\circ }$ are, respectively, 2.441 (2.444), 2.440 (2.445), 2.442 (2.444), 2.444 (2.444), 2.444 (2.444), and 2.442 (2.444) Å, and the lengths of the W–Se bond in ZnO/WSe${}_{2}$ are, respectively, 2.540 (2.544), 2.539 (2.544), 2.541 (2.544), 2.544 (2.544), 2.543 (2.544), and 2.542 (2.544). The length of the W–S (W–Se) bond in ZnO/WS${}_{2}$ (ZnO/WSe${}_{2}$) are slightly larger (smaller) than the original length of the W–S (W–Se) bond in the WS${}_{2}$ (WSe${}_{2}$) monolayer, which is due to the fact that a small lattice mismatch causes small atom rearrangements. When the rotation angles are in the range of 0–300${}^{\circ }$, the layer distances between the two layers in ZnO/WSe${}_{2}$ (ZnO/WSe${}_{2}$) are 2.976, 2.932, 2.964, 3.316, 3.328, and 2.974 (3.084, 3.071, 3.036, 3.375, 3.374, and 3.067) Å, respectively.

**Figure 2:**
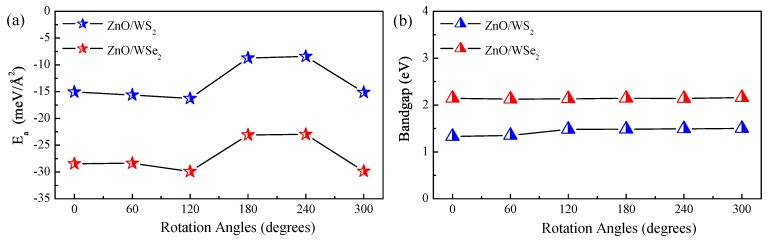
(**a**) Interface adhesion energies and (**b**) bandgaps of ZnO/WS${}_{2}$ and ZnO/WSe${}_{2}$ with different rotation angles.

The relative stability of ZnO/WS${}_{2}$ and ZnO/WSe${}_{2}$ could be compared through a calculation of interface adhesion energy. The interface adhesion energies ($E_{a}$) for ZnO/WS${}_{2}$ (ZnO/WSe${}_{2}$) are defined as

(1) $$E_{a}=\left[E_{\mathrm{ZnO}/{\mathrm{WS}}_{2}\left({\mathrm{WSe}}_{2}\right)}-E_{\mathrm{ZnO}}-E_{{\mathrm{WS}}_{2}\left({\mathrm{WSe}}_{2}\right)}\right]/S$$

where $E_{\mathrm{ZnO}/{\mathrm{WS}}_{2}\left({\mathrm{WSe}}_{2}\right)}$, $E_{\mathrm{ZnO}}$, and $E_{{\mathrm{WS}}_{2}\left({\mathrm{WSe}}_{2}\right)}$ are the total energies for the relaxed ZnO/WS${}_{2}$ (ZnO/WSe${}_{2}$), g-ZnO, and WS${}_{2}$ (WSe${}_{2}$) monolayers. *S* is the top area of the heterostructure. Figure 2a gives the $E_{a}$ values of ZnO/WS${}_{2}$ and ZnO/WSe${}_{2}$ of different rotation angles, and all these six configurations for both ZnO/WS${}_{2}$ and ZnO/WSe${}_{2}$ heterostructures possess negative interface adhesion energies, implying that the formation of these interfaces are exothermic and that these heterostructures could be easily prepared. It is interesting that the varied tendency of the $E_{a}$ values for ZnO/WS${}_{2}$ and ZnO/WSe${}_{2}$ with different rotation angles are almost the same, which is attributed to the similar geometric structures and elemental compositions of these heterostructures. Either ZnO/WS${}_{2}$ or ZnO/WSe${}_{2}$ has a minimum $E_{a}$ value at a rotation angle of 120${}^{\circ }$ in the corresponding heterostructures, indicating that these two heterostructure configurations are the most stable structures in the considered configurations. When the rotation angle is 120${}^{\circ }$, the $E_{a}$ values for ZnO/WS${}_{2}$ and ZnO/WSe${}_{2}$ are respectively −16.28 and −29.92 meV/Å${}^{2}$, within the scope of a vdW $E_{a}$ value of around 20 meV/Å${}^{2}$ [37]. This indicates that ZnO/WS${}_{2}$ and ZnO/WSe${}_{2}$ are vdW heterostructures. Figure 2 shows the varied bandgaps of ZnO/WS${}_{2}$ and ZnO/WSe${}_{2}$ with different rotation angles. The calculated bandgaps for ZnO/WS${}_{2}$ (ZnO/WSe${}_{2}$) of the rotation angles in the range of 0–300${}^{\circ }$ are 1.33, 1.35, 1.48, 1.487, 1.491, and 1.50 (2.14, 2.125, 2.134, 2.15, 2.14, and 2.16) eV, respectively. The bandgaps for ZnO/WS${}_{2}$ and ZnO/WSe${}_{2}$ heterostructures are obviously smaller than the bandgap of the ZnO monolayer and favorable for visible light absorption. The bandgaps of ZnO/WS${}_{2}$ and ZnO/WSe${}_{2}$ heterostructures are almost unchanged when the rotation angles vary, meaning that the rotation component has a negligible impact on the bandgaps of these heterostructures, i.e., the different stacked models will not qualitatively affect our conclusion. Therefore, we could neglect the tiny effect on the electronic structures of heterostructures caused by the rotational component. The following calculations and discussions about the effect of strains on the electronic structures are focused on the ZnO/WS${}_{2}$ and ZnO/WSe${}_{2}$ with the smallest $E_{a}$ value, i.e., ZnO/WS${}_{2}$ and ZnO/WSe${}_{2}$ with the rotation angle of 120${}^{\circ }$.

**Figure 3:**
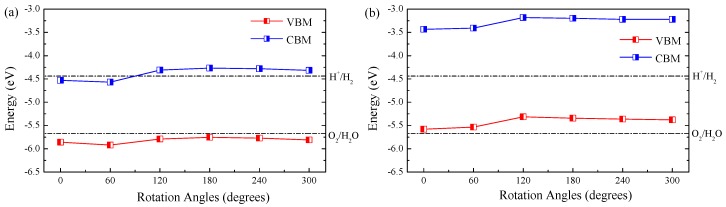
Band alignment of (**a**) ZnO/WS${}_{2}$ and (**b**) ZnO/WSe${}_{2}$ of different rotation angles with respect to the water redox levels.

The suitable bandgap may not always ensure the enhancement of photocatalytic activity. One should also pay attention to band edge alignment in reference to the water redox level. A desired water-splitting photocatalyst must have a VBM lower than the water oxidation level and a CBM higher than the water reduction level. Figure 3 plots the band edge alignment of ZnO/WS${}_{2}$ and ZnO/WSe${}_{2}$ of different rotation angles. The band edge positions of ZnO/WS${}_{2}$ with the rotation angles of 120, 180, 240, and 300${}^{\circ }$ straddle the water redox levels, suggesting that these heterostructures have the ability to act as photocatalysts for the overall water splitting process. For ZnO/WS${}_{2}$ with rotation angles of 0 and 60${}^{\circ }$, the CBM positions are lower than the water reduction level, which make these two heterostructures unfavorable for the spontaneous production of hydrogen. For ZnO/WSe${}_{2}$ with different rotation angles, VBM positions are above the water oxidation level, which causes poor oxygen evolution efficiency.

**Figure 4:**
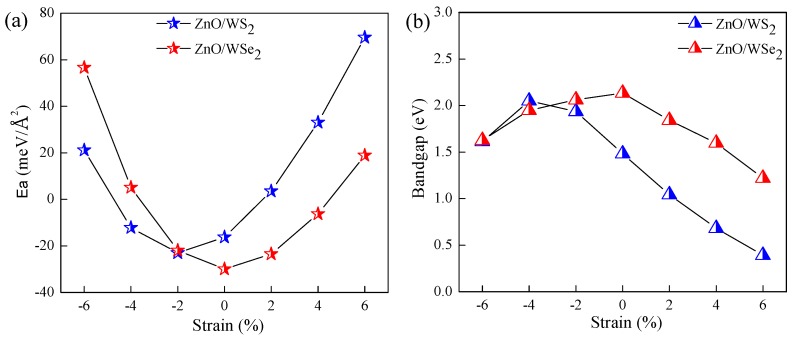
(**a**) Interface adhesion energies and (**b**) bandgaps of ZnO/WS${}_{2}$ and ZnO/WSe${}_{2}$ with different strains.

When two materials with different lattice constants form a heterostructure, the strain will obviously affect the geometry and electronic properties. In addition, many studies report that the electronic and optical properties of 2D materials [38,39,40] could be effectively tuned through the application of strain. The biaxial strain, which is calculated as ϵ = $\left[\left(a-a_{0}\right)/a_{0}\right]$ × 100% (*a* and $a_{0}$ are, respectively, the lattice parameters with and without biaxial strain), is considered to alter the photocatalytic activities of ZnO/WS${}_{2}$ and ZnO/WSe${}_{2}$. Figure 4a indicates that $E_{a}$ values become smaller in the range of ϵ = −6%–−2% but become larger in the range of ϵ = −2%–+6%, which means that the ZnO/WS${}_{2}$ with a strain of −2% is a more stable configuration as compared to these others. The $E_{a}$ values of ZnO/WSe${}_{2}$ become smaller in the range of ϵ = −6%–0 but become larger in the range of ϵ = 0–+6%, implying that the ZnO/WSe${}_{2}$ without strain is energetically more favorable in contrast with g-ZnO. The $E_{a}$ values for ZnO/WS${}_{2}$ of ϵ = −2% and ZnO/WSe${}_{2}$ of ϵ = 0 are, respectively, −22.97 and −29.92 meV/Å${}^{2}$. Hence, these two heterostructures belong to vdW heterostructures. The bandgaps of ZnO/WS${}_{2}$ and ZnO/WSe${}_{2}$ of different strains are depicted in Figure 4b. The bandgaps for ZnO/WS${}_{2}$ of ϵ = −6%–+6% are, respectively, 1.61, 2.05, 1.94, 1.48, 1.04, 0.68, and 0.39 eV, and the bandgaps for ZnO/WSe${}_{2}$ of ϵ = −6%–+6% are, respectively, 1.63, 1.95, 2.06, 2.13, 1.84, 1.60, and 1.22 eV. The bandgaps of ZnO/WS${}_{2}$ become larger in the range of ϵ = −6%–−4% but become smaller in the range of ϵ = −4%–+6%, and the ZnO/WS${}_{2}$ of ϵ = −4% has the largest bandgap. The bandgaps of ZnO/WSe${}_{2}$ become larger in the range of ϵ = −6%–0 but become smaller in the range of ϵ = 0–+6%, i.e., the ZnO/WSe${}_{2}$ without strain has the largest bandgap.

**Figure 5:**
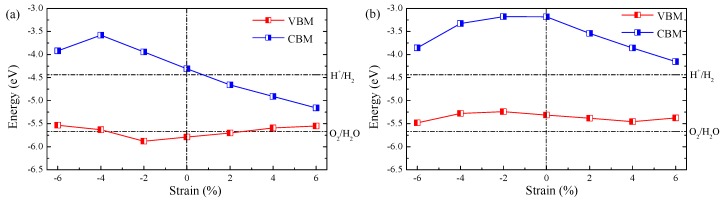
Band alignment of (**a**) ZnO/WS${}_{2}$ and (**b**) ZnO/WSe${}_{2}$ with different strains.

The band edge alignment of ZnO/WS${}_{2}$ and ZnO/WSe${}_{2}$ heterostructures with different strains is given in Figure 5. The band edge positions of ZnO/WS${}_{2}$ heterostructures with ϵ = −2% straddle the water redox levels, implying that these heterostructures are suitable for both hydrogen and oxygen evolution. For ZnO/WS${}_{2}$ heterostructures with ϵ = −6% and −4%, the CBM levels are suitable for hydrogen evolution, but VBM levels are unfavorable for oxygen evolution. While for the case of ZnO/WSe${}_{2}$ heterostructure with ϵ = +2%, though the VBM level is favorable for spontaneous oxygen production, the CBM level is unfavorable for spontaneous hydrogen production. The band edge positions of ZnO/WS${}_{2}$ heterostructures with ϵ = +4% and +6% lie between the water reduction potential and water oxygen potential, which makes these heterostructures unfavorable for over all water splitting process. For the case of ZnO/WSe${}_{2}$ heterostructures with different strains, though the CBM levels are suitable for generating hydrogen, the VBM levels are unfavorable for generating oxygen.

**Figure 6:**
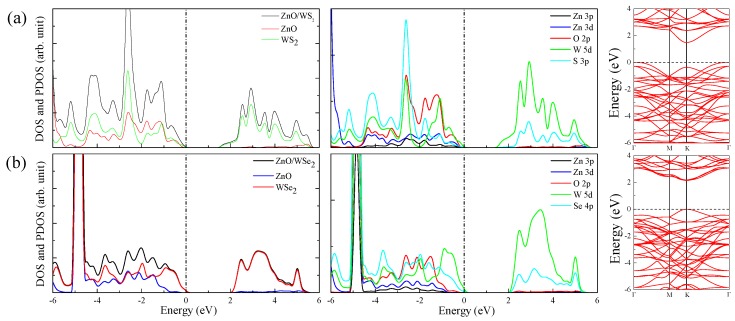
DOS, PDOS, and band structures of (**a**) ZnO/WS${}_{2}$ and (**b**) ZnO/WSe${}_{2}$.

**Figure 7:**
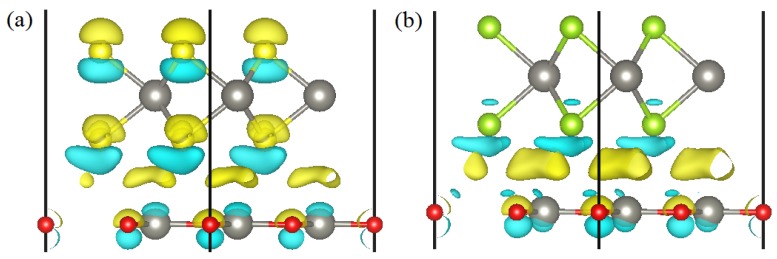
Side views of the charge differences of (**a**) ZnO/WS${}_{2}$ and (**b**) ZnO/WSe${}_{2}$.

The DOS, PDOS, and band structures of ZnO/WS${}_{2}$ and ZnO/WSe${}_{2}$ are shown in Figure 6. The CBM and VBM, respectively, are located at *K* and Γ, suggesting that ZnO/WS${}_{2}$ has an indirect bandgap. The CBM is primarily caused by W 5d orbitals and a small amount of S 3p orbitals, while VBM predominantly consists of W 5d orbitals. The electrons below the Fermi levels are mainly excited from W 5d (O 2p, S 3p) to S 3p (W 5d) orbitals, when the electronic transition of angular momentum selection rules of Δl = ±1 is considered. Figure 7a indicates the electrons in the ZnO layer will migrate to the WS${}_{2}$ layer, which will be helpful for effective separation of photogenerated carriers. Both the CBM and VBM of ZnO/WSe${}_{2}$ are prominently caused by W 5d and Se 4p orbitals and a small amount of O 2p orbitals. After absorbing the photo energy, the electrons in the W 5d (Se 4p) orbitals below the Fermi level will jump to W 5d (Se 4p) orbitals of the conduction band, and only a small amount of electrons jump from W 5d (O 2p) to O 2p (W 5d) orbitals. Figure 7b implies the electrons in the ZnO layer will transfer to the WSe${}_{2}$ layer, which is usually favorable for the effective separation of photogenerated carriers.

**Figure 8:**
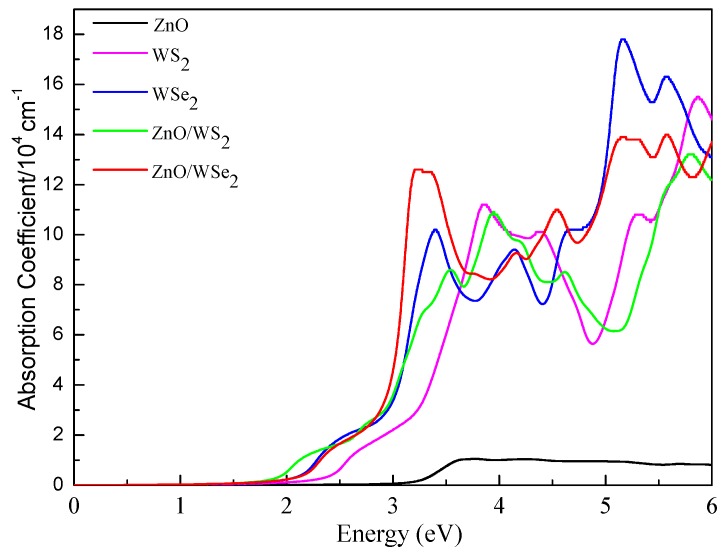
Calculated optical absorption curves of ZnO, WS${}_{2}$, and WSe${}_{2}$ monolayers and of ZnO/WS${}_{2}$ and ZnO/WSe${}_{2}$ heterostructures.

The calculated optical absorption curves for the ZnO, WS${}_{2}$, WSe${}_{2}$ monolayers and the ZnO/WS${}_{2}$ and ZnO/WSe${}_{2}$ heterostructures are depicted in Figure 8. The absorption curve of g-ZnO is limited to the ultraviolet region, whereas WS${}_{2}$ and WSe${}_{2}$ monolayers could absorb visible light and show obvious visible light absorption. Moreover, it is noted that ZnO/WS${}_{2}$ could absorb more visible light as compared to the g-ZnO and WS${}_{2}$ monolayers. The visible light absorption of ZnO/WSe${}_{2}$ is not improved in contrast with the WSe${}_{2}$ monolayer but is obviously improved in contrast with g-ZnO.

## 4. Conclusions

In summary, we perform extensive hybrid density functional calculation to examine the geometric, electronic, and optical properties as well as the band edge alignment of ZnO/WS${}_{2}$ and ZnO/WSe${}_{2}$ heterostructures and consider the possible effect caused by rotation angles and biaxial strains. ZnO/WS${}_{2}$ and ZnO/WSe${}_{2}$ with suitable rotation angles and strains are not difficult to prepare due to the negative interface adhesion energies. The bandgaps of these heterostructures are not obviously affected by the rotation angles, but they are by the strains. The band edge positions render ZnO/WSe${}_{2}$ with different rotation angles and biaxial strains suitable for hydrogen generation but unfavorable for oxygen generation. ZnO/WS${}_{2}$ with suitable rotation angles and strains have appropriate bandgaps for visible light absorbtion and proper band edge alignment for spontaneous water splitting. The charge transfer from the ZnO layer to the WS${}_{2}$ layer will facilitate the separation of photogenerated carriers and improve the photocatalytic activity. These findings imply ZnO/WS${}_{2}$ is a promising water-splitting photocatalyst.
